# A Call for Consensus: A Narrative Review of GPS-Based External Training Load Monitoring in Male Youth Soccer Players

**DOI:** 10.3390/sports14040152

**Published:** 2026-04-14

**Authors:** Krisztián Havanecz, János Matlák, Ferenc Ihász, Gábor Géczi, Bence Kopper, Sándor Sáfár, Gábor Schuth

**Affiliations:** 1 Training Theory and Methodology Research Center, Hungarian University of Sports Science, 1123 Budapest, Hungary; safar.sandor@tf.hu; 2 Department of Kinesiology, Hungarian University of Sports Science, 1123 Budapest, Hungary; matlak.janos@tf.hu (J.M.); kopper.bence@tf.hu (B.K.); 3 Department of Health Promotion and Exercise Science, Széchenyi István University, 9026 Győr, Hungary; ihasz.ferenc@sze.hu; 4 Department of Sport Management, Hungarian University of Sports Science, 1123 Budapest, Hungary; geczi.gabor@tf.hu; 5 Department of Sport Medicine and Sport Science, Hungarian Football Federation, 1112 Budapest, Hungary; schuth.gabor@mlsz.hu

**Keywords:** GPS, IMU, monitoring, player load, review, total distance, training load, training load monitoring, youth soccer

## Abstract

Background: Global positioning system (GPS) technology is widely used to quantify external training load (ETL) in youth soccer. Despite its extensive application in training and match contexts, considerable heterogeneity is present in the selection, definition, and interpretation of GPS-derived variables, limiting comparability between studies and practical implementation by coaches. Objective: This narrative review aimed to summarize and critically evaluate the current literature on GPS-based ETL monitoring in youth soccer players, with a focus on commonly used variables, methodological considerations, and practical applications in training and match contexts. Methods: A narrative literature search was conducted using PubMed, SPORTDiscus, and Scopus databases. Peer-reviewed studies published in English between the years of 2012 and 2025 were included. Data were extracted on participant characteristics, GPS technology, monitored ETL variables, and contextual settings. Results: The 34 reviewed studies primarily reported total distance (TD; m), high-speed running distance (HSR; m), sprint distance (SD; m), distance per minute (m·min^−1^), peak speed (km·h^−1^), and acceleration- and deceleration-based (ACC, DEC; count) ETL variables. Substantial variability was observed in speed thresholds, acceleration definitions, and data processing methods. Positional roles, training formats (e.g., small-sided games), and seasonal phase influenced ETL demands, although methodological inconsistencies limited cross-study comparisons. Conclusion: GPS technology provides valuable insights into the ETL demands of youth soccer. The lack of standardized variable definitions and thresholds remains a major limitation. Greater methodological consistency and clearer reporting standards are required to enhance the practical usefulness of GPS monitoring for coaches in youth soccer.

## 1. Introduction

The quantification of external training load (ETL) has become a fundamental component of training load (TL) monitoring in modern soccer. In elite youth contexts, where players are exposed to progressively increasing physical demands alongside ongoing growth and maturation, objective assessment of training and match loads is considered essential for supporting long-term athlete development and evidence-based coaching practice [1,2]. Advances in wearable tracking technologies, namely global positioning system (GPS)-based devices, have substantially enhanced the ability of practitioners to monitor the ETL demands imposed on youth soccer players during both training and match [3]. GPS is a satellite-based positioning system within the global navigation satellite system (GNSS), enabling spatial tracking of outdoor movement. In sport tracking devices, the term “GPS” is often used generically as an umbrella term, although many tracking systems operate using multi-GNSS positioning.

The use of GPS technology in team sports emerged in the early 2000s, initially within elite adult populations, as a response to the need for objective and continuous monitoring of players’ movement demands [4]. Over the past two decades, improvements in satellite technology, sampling frequency, and data processing algorithms have markedly increased the accuracy and practicality of GPS devices. Consequently, GPS-based monitoring has transitioned from a research-oriented tool to a routine component of TL monitoring systems in professional soccer clubs and, more recently, elite youth soccer academies [5,6]. In the context of this review, elite youth academies refer to structured development programs affiliated with professional soccer clubs or national training centers that systematically monitor TL and player development. As soccer academies increasingly adopt similar physical development pathways aligned with professional standards, GPS monitoring has become a widely accepted method for quantifying training and match demands across different age groups [7,8].

ETL refers to the physical work accomplished by the athlete, independent of the internal physiological or perceptual responses elicited by that work [9]. In soccer research and applied practice, ETL is commonly quantified using GPS-derived metrics such as total distance (TD; m), high speed running (HSR; m), sprint distance (SD; m), distance covered per unit of time (m·min^−1^), peak running speed (km·h^−1^), accelerations (ACCs; count), and decelerations (DECs; count) [10,11]. These variables are widely used to describe match demands, evaluate training drills, and monitor load accumulation across training microcycles in youth soccer [2,3,6,8].

The widespread adoption of GPS technology in soccer is underpinned by extensive research demonstrating acceptable levels of validity and reliability for the measurement of running-based activities, such as for TD and average running speed (~1–4% coefficient of variation, CV), with moderately high variability for higher speed running-based metrics (~4–10% CV) [12,13]. Early validation studies reported that GPS devices with higher sampling frequencies (e.g., ≥10 Hz) provide more accurate estimates of distance, speed, and high-intensity running compared to earlier low-frequency systems [4,12]. Subsequent investigations confirmed that GPS units show good to excellent reliability for TD and HSR during soccer-specific movements [13,14]. In youth cohorts, GPS technology has similarly demonstrated acceptable reliability for commonly used ETL variables, supporting its application in academic settings [10]. However, the validity of acceleration- and deceleration-derived metrics appears to be primarily influenced by technological and measurement-specific factors (e.g., high-intensity acceleration distance), rather than by inherent limitations of the constructs themselves [12,13]. Evidence suggests that GPS-based systems operating at 10 Hz may show reduced accuracy, when quantifying instantaneous and maximal ACC, as well as the frequency of high-intensity ACC events (e.g., >3 m·s^−2^), with coefficients of variation typically ranging between ~10–20%, whereas acceptable levels of accuracy have been reported for lower ACC zones (CV ~3–8%) [12,13]. Hence, the precision of these metrics is highly dependent on these technological capabilities. In this regard, inertial measurement unit (IMU) sensors have been proposed as a complementary approach for capturing rapid changes in movement. In soccer, IMUs are commonly integrated within GPS tracking units and provide mechanical load indicators (e.g., accelerations, decelerations) derived from triaxial accelerometer, magnetometer, and gyroscope microsensors. In addition, previous studies have applied standardized data quality criteria for analyses, including a minimum number of connected satellites (more than six satellites) and the average horizontal dilution of precision (HDOP < 1 a.u.), all of which are considered critical factors for ensuring the validity of GPS-derived variables [1]. Additionally, the manufacturer’s device software and firmware version update may influence the collected GPS data. According to Bourdon et al. (2017), differences in device models, data processing algorithms, and software or firmware updates may substantially influence derived external load metrics [6]. As these proprietary processing procedures are rarely fully disclosed, IMU-based variables may differ across systems or software versions (e.g., registration time of an acceleration event), even when identical thresholds are applied. These limitations are particularly relevant in youth players, whose movement patterns and neuromuscular capacities differ from those of adults.

In adolescent athletes, appropriate ETL monitoring is particularly important due to the interaction between training stress, biological maturation, and injury risk [1,15]. During periods of rapid growth, youth players may experience transient reductions in movement efficiency and an increased likelihood of non-contact injuries related to overload [15]. GPS-derived ETL data provide practitioners with objective information to guide training prescription and manage weekly TL distribution (exposure to high-intensity running and mechanical stress). Furthermore, GPS monitoring allows coaches to evaluate whether training stimuli adequately prepare youth players for the demands of match circumstances, although athlete development is prioritized in youth environments [15]. This is especially relevant in elite academies, where training content must balance physical development with technical and tactical objectives. As such, GPS-based ETL monitoring has become an integral component in elite youth soccer [5,6].

Despite its extensive application, the scientific literature examining GPS-based ETL monitoring in elite youth soccer is characterized by substantial methodological heterogeneity. Studies differ considerably in terms of GPS device characteristics (e.g., dimensions of the sensors), sampling frequencies, data filtering procedures, and the selection of ETL variables [6]. One of the most prominent sources of inconsistency relates to the use of a speed threshold to categorize running intensity. Absolute speed zones are frequently applied across youth age groups, despite well-documented differences in physical capacity, maturation status, and positional demands [10,16,17]. As a result, identical ETL values may represent substantially different mechanical and physiological stress between players.

Similar methodological challenges exist in the quantification of ACCs and DECs. Variability in threshold selection, minimum event duration, and data processing algorithms limits the comparability of inertial measurement unit (IMU)-derived metrics across studies [18,19]. For example, Varley et al. (2017) demonstrated that modifying the minimum effort duration criterion substantially altered the number of detected high-intensity acceleration events, despite an identical intensity threshold being applied previously [20]. Furthermore, a recent study from Australian football showed that the application of different data processing methods to identical global navigation satellite system datasets resulted in large differences in the total number of ACCs, highlighting the sensitivity of acceleration metrics [21]. Although ACC and DEC variables are often proposed as sensitive indicators of mechanical training load in soccer, particularly during changes in direction, their interpretation in youth populations remains complex due to inconsistent methodological approaches [22].

In addition to methodological issues, contextual factors, such as playing position, training format, and seasonal phase, substantially influence ETL profiles. Match play typically elicits higher absolute ETL than training sessions; however, specific training formats, such as small-sided games, may replicate or exceed match demands for intensity-based variables depending on pitch size, player numbers, and rule constraints [7,23]. In this context, caution is warranted when interpreting acceleration- and deceleration-based metrics derived from GPS technology with integrated IMUs. Beyond differences in sampling frequency and algorithms, the underlying measurement principles differ. GPS-derived ACCs are inherently displacement-dependent and typically require a minimal horizontal movement of 1–2 m to be registered by the sensor, whereas IMU-based ACCs may be triggered by rapid changes in body posture. Recognizing these contextual influences is essential for coaches seeking to manipulate TL while maintaining technical and tactical relevance.

Although several research articles have examined GPS-derived ETL monitoring in adult soccer or across multiple team sports [5,6,8,24,25,26,27], focused syntheses addressing elite youth soccer populations remain limited. A previous systematic review has synthesized match running performance measures in youth soccer players, highlighting positional and age-related differences in TD and speed zone metrics [28]. However, since then, numerous studies using these technologies have been published. Despite the widespread use of GPS-derived ETL metrics in youth soccer, considerable methodological heterogeneity exists regarding variable definitions, speed thresholds, and data processing procedures, which complicates comparisons across studies and limits the development of standardized monitoring approaches. Given the unique developmental, physiological, and organizational characteristics of youth academies, a targeted evaluation of the available literature is warranted. In the context of the present review, the term “consensus” refers to the development of more harmonized methodological practices and reporting standards for GPS-derived ETL monitoring in youth soccer.

Therefore, this narrative review aimed to synthesize and critically evaluate the international literature on GPS-based ETL monitoring in elite youth soccer players. This review sought to (i) summarize the GPS-derived ETL variables most frequently reported in youth soccer research, (ii) identify key methodological inconsistencies related to data collection and reporting, and (iii) discuss practical implications for practitioners working in elite youth soccer environments.

## 2. Materials and Methods

### 2.1. Review Design

This study was conducted as a narrative review aimed at synthesizing and critically evaluating the existing literature on GPS-based ETL monitoring in youth soccer players. A narrative approach was selected to allow flexibility in summarizing methodological trends, contextual influences, and practical implications relevant to coaching.

### 2.2. Literature Search Strategy

A comprehensive literature search was performed using PubMed, SPORTDiscus (EBSCOhost), and Scopus databases. Additionally, relevant studies were identified through manual reference screening. The search strategy was designed to identify peer-reviewed studies investigating GPS-derived ETL variables in youth soccer players. The search was restricted to articles published in English between January 2012 and December 2025. Search terms included combinations of keywords related to soccer, youth athletes, GPS technology, and ETL monitoring. The following search string was applied across databases with minor adaptations according to database-specific syntax: (soccer OR football) AND (youth OR adolescent OR academy) AND (GPS OR “global positioning system”) AND (“external load” OR “external training load” OR “training load”). In Scopus, the following field-specific query was used: TITLE-ABS-KEY (soccer OR football) AND TITLE-ABS-KEY (youth OR adolescent OR academy) AND TITLE-ABS-KEY (GPS OR “global positioning system”) AND TITLE-ABS-KEY (“external load” OR “external training load” OR “training load”). Database-specific filters were applied where appropriate to refine the search results. The initial search covered studies published between 2012 and 2025, reflecting the widespread adoption of modern high-frequency GPS technology in applied soccer research. Duplicate records retrieved from multiple databases were removed prior to the screening process. Additional relevant studies were identified through manual screening of reference lists from included articles.

### 2.3. Eligibility Criteria

Studies were included if they met the following criteria:

- Participants were male soccer players (U11–U23) competing in football (pre)-academies or national development programs;
- GPS technology was used to quantify external training load during training sessions and/or matches;
- Studies reported at least one GPS-derived ETL variable (e.g., total distance, high-speed running distance, sprint distance, total distance per minute, peak speed, accelerations, and decelerations);
- Only open-access articles were considered to ensure transparency and accessibility of the reviewed literature for practitioners and researchers;
- The study was published in a peer-reviewed journal and written in English.

Studies were excluded if they focused exclusively on adult players, recreational athletes, or if GPS technology was not used to quantify ETL, or sports other than soccer.

### 2.4. Study Selection and Data Extraction

**Figure 1:**
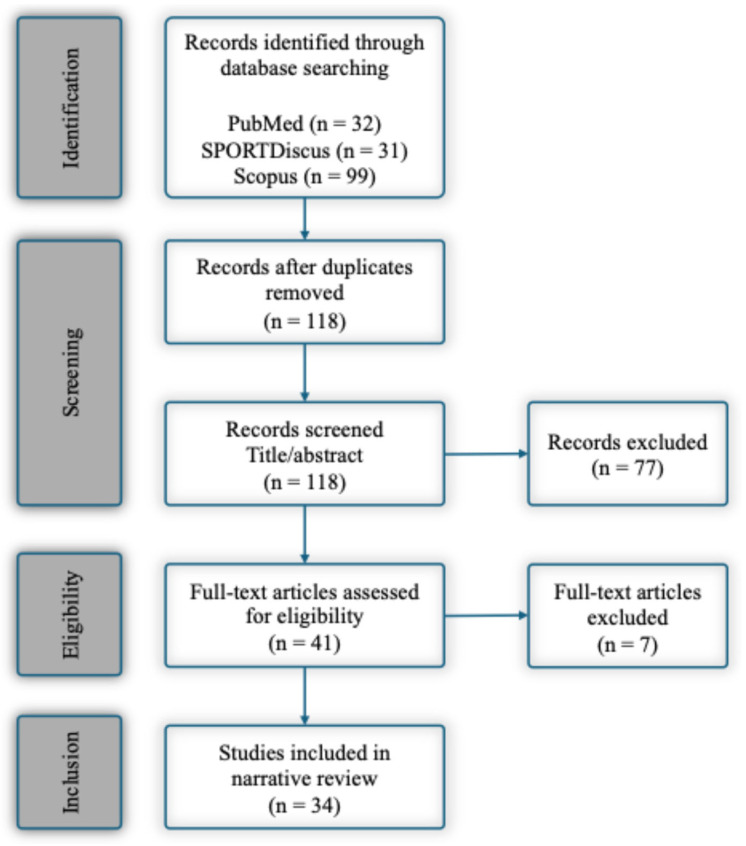
PRISMA flow diagram of the literature search and study selection process.

Titles and abstracts identified through the database search were independently screened for relevance by two reviewers. Discrepancies were resolved through discussion until consensus was reached. Full texts of potentially eligible studies were subsequently assessed against the inclusion criteria. For each included study, data were extracted on participant characteristics, GPS technology and sampling frequency, monitored ETL variables, contextual settings (activity type, specific training format), and key findings. Given the narrative nature of the present review, a formal methodological quality scoring tool was not applied. However, the methodological characteristics of the included studies were qualitatively evaluated based on aspects relevant to GPS-derived ETL research, including study design, participant characteristics, monitoring environment (trainings, matches), GPS technology specifications (frequency, integrated inertial sensors), and well-defined ETL variables. The study selection process is summarized in a PRISMA-style flow diagram (Figure 1).

### 2.5. Data Synthesis

The extracted data were synthesized qualitatively. Studies were grouped according to three main analytical categories: (i) commonly reported ETL variables, (ii) methodological characteristics of GPS monitoring (e.g., speed and acceleration thresholds, variable definitions, and reporting practices), and (iii) contextual factors influencing ETL, including playing position, training format, and seasonal phase.

### 2.6. Methodological Considerations

Given the narrative design of the review, the primary objective was not to provide an exhaustive or statistically pooled summary of all available evidence, but rather to identify common practices, methodological inconsistencies, and applied trends within the youth soccer literature. This approach was considered appropriate for addressing practical questions relevant to researchers and practitioners studying and/or working in youth soccer.

## 3. Results

### 3.1. Study Characteristics

The database search and subsequent manual screening process resulted in the inclusion of 34 empirical studies for qualitative synthesis. The included studies investigated GPS-based ETL monitoring in male youth soccer players, primarily within soccer academies. The majority of studies focused on players aged between the under-11 and under-23 age categories. Study designs were predominantly observational, employing longitudinal or repeated measures approaches to assess ETL across training sessions, match play, or both. Sample sizes varied across studies, reflecting differences in academy structure, monitoring duration, and competitive level. Several investigations monitored players across multiple weeks or seasonal phases, enabling the examination of training microcycles, positional demands, and contextual influences on ETL. The included studies were analyzed with particular focus on three main aspects: (i) commonly reported GPS-derived ETL variables, (ii) methodological characteristics of GPS monitoring, (iii) and contextual factors influencing ETL demands in youth soccer (e.g., training or match).

### 3.2. GPS Technology and Monitoring Protocols

All included studies utilized GPS-based tracking systems to quantify ETL. Sampling frequencies ranged from 1 to 18 Hz, with more recent studies predominantly employing high-frequency devices (≥10 Hz) [8,24,25,26,27,29,30,31,32,33,34,35,36,37,38,39,40,41,42,43,44,45,46,47,48,49,50]. In some investigations, GPS data were extended with inertial measurement unit (IMU)-derived metrics to capture micromovements, such as ACCs, DECs, and PL [8,24,25,26,27,29,33,34,36,37,38,39,40,41,42,43,44,45,46,47,51,52,53,54]. Devices were typically worn between the scapulae using a manufacturer-provided sports vest. Although most studies reported basic device characteristics, there was considerable variability in the reporting of data filtering procedures, signal processing methods, and variable calculation algorithms.

### 3.3. Contextual Settings and External Training Load Variables

**Figure 2:**
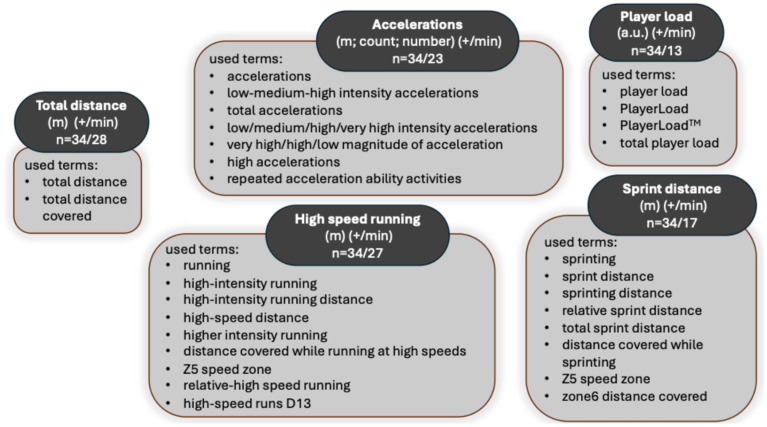
Heterogeneity in the definition of commonly reported ETL variables in youth soccer.

ETL was assessed across a range of soccer-specific contexts. Match-play demands were commonly analyzed during competitive fixtures [16,30,31,32,33,36,37,39,41,42,48,51,52], while training-based studies examined whole-session ETL, drill-specific demands, or small-sided games [8,24,34,45,46,47,49], and both training and match were studied at the same time [25,26,27,29,35,38,40,44,45,50,53,54,55,56]. Several studies explored positional differences, highlighting distinct ETL profiles associated with tactical roles. Across the included studies, TD and HSR were the most consistently reported ETL variables, while ACCs, SD, and PL were also frequently reported (see Figure 2). However, speed thresholds used to define these variables varied substantially. For example, several speed thresholds were reported regarding high-intensity running distance: >14.4 km·h^−1^ [36,41], >18 km·h^−1^ [49], 18.0–20.9 km·h^−1^ [39,55], >19.1 km·h^−1^ [35], 19.8–25.1 km·h^−1^ [8,26,27,47], 20–25 km·h^−1^ [44], and 19.8–25.2 km·h^−1^ [41,54]. ACC- and DEC-based metrics were included in several studies, although their operational definitions and threshold criteria differed across studies, resulting in the use of lower-intensity ACCs (~1.5–2.0 m·s^−2^) and DECs (~−1.5–−2.0 m·s^−2^) [47], both lower-intensity ACCs and DECs [24,26,27,38,54,55], in some studies, higher-intensity ACCs (~2.5–3.0 m·s^−2^), and DECs (<−2.5–−3.0 m·s^−2^) [8,33,35,37,39,42,45,47], in others, and multi-level zone classifications in several cases [25,29,41,49]. Most studies used absolute speed thresholds, whereas only a limited number of relative approaches were reported [16].

Table 1 summarizes 34 studies examining GPS-based ETL monitoring in youth soccer players.

To provide a more structured overview of methodological heterogeneity, Table 2 summarizes the reported threshold ranges used to define high-speed running, sprints, accelerations, and decelerations across the included studies.

In addition to variability in reported ETL variables, Figure 2 represents the substantial heterogeneity that was observed in the absolute speed thresholds and definitions across studies. This variability highlights the lack of standardized reporting practices and complicates direct comparisons (cross-study) of GPS-derived ETL variables in youth soccer research.

## 4. Discussion

The present narrative review aimed to synthesize and critically evaluate the existing literature on GPS-based ETL monitoring in youth soccer players. The main findings indicate that GPS technology is widely applied within youth soccer academies to quantify running-based and mechanical training load. However, substantial heterogeneity exists in the selection, definition, and operationalization of ETL variables.

As a body of evidence, the 34 included studies should be interpreted with caution. Most investigations employed observational or repeated-measures designs, which are valuable for describing ETL patterns, but do not allow causal inference regarding the relationships between external load, adaptation, or injury-related outcomes. In addition, software/firmware versions were rarely reported, despite the likelihood that updates in manufacturer-specific processing algorithms may influence derived ETL metrics and reduce reproducibility across studies [6]. The included studies also varied in sample size, age-group categorization, and monitoring duration, ranging from one-time observations and tournament-based datasets to full-season analyses, which constrains the generalizability of findings across different academy environments. Furthermore, differences in device manufacturers and proprietary algorithms may be particularly relevant for acceleration- and deceleration-based processing procedures, which may alter the number of detected ACC and DEC events [18,19,20,21].

**Figure 3:**
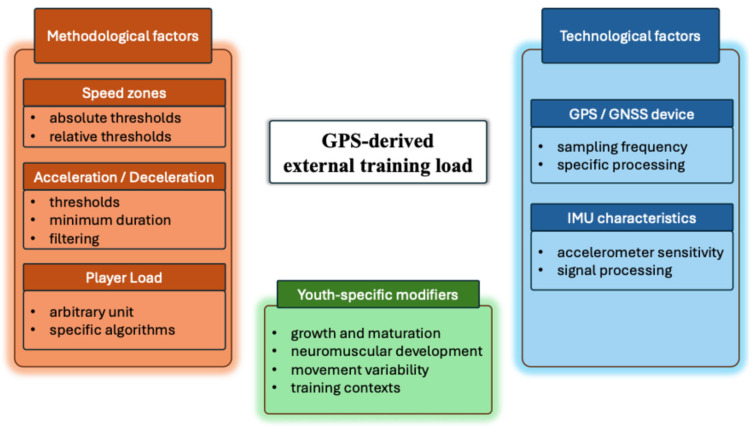
Conceptual framework illustrating methodological and contextual factors influencing the interpretation of GPS-derived ETL in youth soccer.

The main methodological, technological, and youth-specific factors influencing the interpretation of GPS-derived ETL metrics in youth soccer are summarized in Figure 3. The figure is presented in the current section to support the interpretation of these interacting factors in relation to the reviewed findings. The conceptual framework highlights that ETL values are not determined solely by physical activities, but may also be influenced by device characteristics, data processing procedures, speed zones, and developmental factors such as growth and maturation of the athletes.

Across the included studies, TD emerged as the most consistently reported GPS-derived ETL variable, reflecting its simplicity and robustness across different devices and contexts [8,10,24,26,27]. Distance per minute (m·min^−1^) was also frequently used to account for variations in exposure time. HSR and SD were commonly reported, although the thresholds used to define these variables varied markedly between studies. Several investigations adopted absolute speed thresholds to categorize running intensities, despite clear evidence that physical capacity and maximal running speed differ substantially across youth age groups and maturation stages [10], but rather used the default settings proposed by the manufacturer. Other studies employed relative approaches based on maximal aerobic speed, anaerobic speed reserve, or maximal sprint speed, which may better reflect the individual physiological demands imposed on developing players [16]. Maximal aerobic speed (MAS) is closely linked to aerobic capacity and may therefore be useful for individualizing training intensity during high-intensity running. Anaerobic speed reserve (ASR) represents speed profiles and facilitates comparisons between players. Maximum sprint speed (MSS) highlights the athlete’s neuromuscular capacity and allows comparison between different positional plays. In youth athletes, differences in biological age and the timing of peak height velocity (PHV) can substantially influence physical development and match running performance. Previous studies have shown that maturity status may affect the high-intensity running and sprint performance independently of chronological age [17,37]. Players in the pre-PHV stage exhibit lower maximal speed and neuromuscular capacities, whereas circa-PHV periods are often associated with transient reductions in movement efficiency and coordination due to rapid growth and more focus on hormonal factors, such as strength development. In contrast, post-PHV athletes generally demonstrate greater sprint capacity and explosive power output. Consequently, identical absolute speed thresholds may represent different physiological loads among players at different stages of maturation. Thus, the coexistence of absolute and relative speed-zone frameworks highlights a lack of consensus for quantifying running intensities in youth soccer.

Acceleration- and deceleration-derived ETL variables were included in several studies, reflecting increased interest in the mechanical training load demands. Nevertheless, marked variability was observed in the thresholds (ACCs > 1.5 m·s^−2^ to >3.0 m·s^−2^; DECs < −1.5 m·s^−2^ to <−3.0 m·s^−2^), minimum duration criteria (0.2–0.6) [20], and filtering procedures used to identify IMU-based events [18,22]. Such methodological diversity complicates interpretation in youth populations, where neuromuscular development and movement efficiency are still ongoing processes.

Nevertheless, the lack of a standardized intensity threshold does not invalidate the use of GPS-derived ETL monitoring in youth soccer but rather redefines its primary scientific and applied purpose. The observed heterogeneity in speed zones, acceleration thresholds, and composite metrics reflects not only methodological inconsistency but also the complex and dynamic nature of youth soccer environments, where growth and maturation interact with external load exposure. Also, it is worth mentioning that ACC thresholds are influenced by the initial running speed, as higher ACCs can be executed from standing compared to higher entry speeds [57]. The validation reports are also available in youth soccer environments. Previous research has demonstrated acceptable reliability for distance- and speed-based GPS variables, when higher sampling frequencies are used (e.g., 10 Hz) [13,14]. In contrast, the validity and reliability of ACCs and DECs metrics appear more sensitive to device specifications and data processing methods [18,19]. IMU sensors are operating at a higher sampling rate (100 Hz), providing more accuracy of micromovements.

Beyond methodological inconsistency, the heterogeneity in ETL definitions may also have practical implications for players’ health. Different absolute thresholds for high-speed running, sprint, or ACCs events may lead to inconsistent estimations of ETL exposure and potentially influence the interpretation of fatigue or non-contact injury risk. Furthermore, such variability may complicate the development of load–injury models [58], as heterogeneous definitions limit the comparability of datasets across studies. This issue may be relevant in youth soccer, where growth and maturation processes influence movement kinematics and the interpretation of GPS-derived indicators.

Several studies reported PL as a manufacturer-specific IMU-derived mechanical training load data from a triaxial accelerometer [24,25,26,27,29,34,36,38,42,45,48,53]. However, this variable represents an arbitrary unit derived from triaxial accelerometer data, and its calculation is dependent on manufacturer-specific algorithms and data processing procedures. Although PL is commonly described as the vector magnitude of changes in ACC across the three orthogonal axes (XYZ) [59,60], variations in differentiation, filtering, and integration procedures across manufacturers are rarely fully disclosed. Hence, identical PL values obtained from different GPS–IMU systems may not be directly comparable. This limitation is particularly relevant in youth soccer, where movement patterns, neuromuscular control, progressive load, and ACC profiles differ substantially across developmental stages [15]. As a result, PL should be interpreted with caution and primarily used for within-player comparisons, rather than cross-study benchmarking.

Contextual factors, including training format, playing position, periodization, development stage, and environmental changes, may influence ETL profiles. Based on previous findings, match play consistently elicited higher absolute ETL values compared to training sessions [7]. Although specific training formats like small-sided games were capable of replicating or exceeding match demands for certain IMU-derived variables, the dependency of pitch dimensions, player numbers, and imposed constraints was influencing these factors [7,23,24]. These findings emphasize the importance of contextualizing GPS-derived ETL data, rather than relying solely on absolute values. Positional differences in ETL were also evident, with wide and midfield players typically exposed to greater running demands than central defenders during matches [10,16].

### 4.1. Limitations

Several limitations must be addressed. The majority of included studies employed observational designs, limiting causal inference regarding training load and performance or injury outcomes. Methodological heterogeneity in GPS technology, data processing, and variable definitions across studies restricts direct comparison. Furthermore, relatively few investigations have examined long-term longitudinal changes in ETL across multiple developmental stages within the same cohort.

Another limitation relates to the absence of a formal methodological quality assessment or risk-of-bias evaluation, which reflects the narrative design of the present review. Furthermore, only open-access articles were included, which may have restricted the breadth of the available evidence. Although multiple databases were used in the revised search strategy, some relevant studies may still have been missed due to differences in database coverage.

Lastly, an important limitation relates to the interpretation of speed and acceleration thresholds in youth soccer. Although several studies propose specific threshold values, these may vary substantially across age groups, maturation stages, and methodological approaches. Consequently, it remains uncertain whether universal threshold values can be established for youth populations or whether the observed variability reflects the inherently heterogeneous nature of developing athletes.

Future research should prioritize standardized reporting frameworks for GPS-derived ETL variables in youth soccer, including explicit justification of speed and acceleration thresholds. Longitudinal designs examining the interaction between maturation and ETL exposure would provide valuable insights for evidence-based youth development programs.

### 4.2. Practical Implications

From an applied perspective, GPS-based ETL monitoring provides valuable objective information to support practitioners regarding training prescription, individual ETL management, and long-term athlete development, while avoiding constant high overload, causing an increased rate of non-contact injuries in youth soccer. Given the interaction between training load, growth, and injury risk during adolescence, systematic monitoring of running and mechanical demands may assist practitioners in managing exposure to high-intensity activities and cumulative load [1,15]. However, the findings of this review suggest that practitioners should be cautious when comparing ETL values across studies or applying default speed thresholds within their own training environments. Greater emphasis should be placed on within-team consistency, transparent variable definitions, and the use of individualized speed thresholds where feasible.

Although the present review did not consider internal training load-based (ITL) variables, it is also suggested to integrate heart rate-derived and rate of perceived exertion measures, which may further enhance the interpretability of monitoring data and provide reliable information about the young players’ physiological adaptation. Importantly, ETL metrics do not replace other monitoring dimensions (e.g., heart rate, subjective questionnaires), and the lack of standardized speed thresholds cannot be compensated for by the isolated inclusion of ITL indicators. Consequently, interpreting ETL variables without physiological context may lead to incomplete interpretations of fatigue, adaptation to stimulus, or non-contact injury risk. As such, integrating ETL (locomotor and/or mechanical load) with ITL (metabolic and/or physiological load) indicators (e.g., heart rate-derived metrics, rating of perceived exertion) may provide a more comprehensive understanding of the training stimulus in youth athletes where growth and maturation may influence the relationship between ETL and ITL [6,9,24,25,26,27].

Furthermore, the youth academy system may further influence the interpretation of GPS monitoring. Characteristics such as player rotation across age groups, positional changes across development stages, and fluctuating competitive demands address broader structural factors that may influence the interpretation of GPS monitoring data.

Lastly, the heterogeneity highlighted in Table 1 and Figure 2 demonstrated that the lack of standardized definitions of variables and speed-band definitions represents a fundamental methodological limitation in youth soccer TL research. Identical ETL constructs, such as high-speed running or sprint running distance, are operationalized using markedly different absolute speed thresholds across studies, frequently determined by manufacturer-specific default settings rather than physiologically or developmentally justified criteria. These findings support the need for more transparent methodological reporting, which may provide an important foundation for the future development of consensus-based approaches in youth soccer. Establishing standardized bands for running speed and ACC intensity would improve cross-study comparability, facilitate data sharing between academies and research groups, and enhance the interpretability of GPS-derived ETL metrics across developmental stages.

To further operationalize the present “call for consensus”, a reporting checklist is proposed for future studies examining GPS-derived external training load in youth soccer:

(i) GPS/GNSS device characteristics: manufacturer name, model, sampling frequency, and software/firmware version used for data collection and processing;
(ii) IMU characteristics integrated into GPS: accelerometer sampling rate, the signal processing approach used to derive IMU-based variables;
(iii) Speed threshold framework: whether absolute or relative thresholds were applied, the exact numerical thresholds used, and the physiological or practical rationale for the selection;
(iv) Acceleration/deceleration criteria: threshold values, minimum effort or dwell time, and filtering or detection procedures used to identify acceleration and deceleration events;
(v) Data quality and processing criteria: minimum number of connected satellites, HDOP (although there is no standardization behind it) or equivalent signal-quality thresholds, and any criteria used to exclude invalid files (e.g., indoor activity), and details regarding filtering, smoothing, export settings, event detection procedures, and treatment of missing or invalid data;
(vi) Participant maturation status: the method used to estimate biological age or maturity offset (e.g., PHV-related approach), or an explicit acknowledgement that maturation was not assessed;
(vii) Variable definitions and units. clear operational definitions of each reported external training load variable, including the unit of measurement (e.g., meter, count, km·h^−1^, m·s^−2^, arbitrary units), and explicit clarification of whether values refer to absolute, relative, or time-normalized outputs;
(viii) Manufacturer-specific derived variables: explicit identification of variables derived from proprietary algorithms (e.g., PlayerLoad), including the reported unit, a brief definition and cited source, and a statement acknowledging that direct comparison across systems may be limited;
(ix) Monitoring context: whether external training load was quantified during matches, full training sessions, field tests, or drill-specific tasks, and whether analyses were based on whole-session, phase-specific, or relative outputs.

## 5. Conclusions

This narrative review highlights the widespread use of GPS technology for monitoring external training load. While TD and HSR are consistently reported, substantial methodological heterogeneity exists in the definition and application of speed and ACC thresholds. Such variability limits cross-study comparability and challenges the interpretation of external load in developing athletes. Greater standardization and transparent reporting are required to enhance the practical and scientific value; thus, future research and applied practice would benefit from the development of consensus-based methodological standards for defining and reporting GPS-derived ETL variables in youth soccer.

## Figures and Tables

**Table 1:** Characteristics of the included studies.

Study	Sample/Age Group/Design	Settings	Tracking Technology	ETL Variables Reported
Mendez-Villanueva et al. (2013) [16]	N = 103, highly trained youth soccer players U13–U18; 42 matches	match first and second half	GPSports SPI Elite^TM^; 1 Hz (Canberra, Australia)	speed zone 1: ≤60% of MAS; speed zone 2: 61–80% of MAS; speed zone 3: 81–100% of MAS; speed zone 4: 101% of MAS to 30% of ASR; speed zone 5: ≥31% of ASR
Barron et al. (2016) [51]	N = 61, well-trained sub-elite youth soccer players; 14 games in-season	match	Minimax Catapult, 5 Hz, integrated IMU 100 Hz (Melbourne, Australia)	repeated acceleration ability activities (>1.5 m·s^−2^): total bouts (n), efforts per bout (n), recovery per effort (s), recovery per bout (s)
Saward et al. (2016) [52]	N = 263, elite male youth soccer players from 3 professional academies (U9–U19); three soccer seasons; 988 matches	match	GPSports SPI Elite^TM^; 1 Hz, integrated IMU 100 Hz; 5 Hz GPS (N/A), integrated IMU 100 Hz (Canberra, Australia)	total distance (m·h^−1^), peak 10–20 m flying speed (m·h^−1^), low-speed distance (<45%, m·h^−1^), high-speed distance (≥45%, m·h^−1^), sprinting distance (≥75%, m·h^−1^)
Beenham et al. (2017) [53]	N = 40; high level youth soccer players; 3 small-sided games, 6 matches	training + match	Catapult Minimax X3, 5 Hz GPS, integrated IMU 100 Hz (Melbourne, Australia)	accumulated player workload per minute, individual axial loads per minute (XYZ axes)
Gómez-Carmona et al. (2018) [29]	N = 20, national-level soccer players (U18); 229 statistical analysis units (37 competition data from 4 matches; 192 recordings from 4 training sessions)	training + match	RealTrack Systems WIMU^TM^, 10 Hz, integrated IMU 100 Hz (Almería, Spain)	total distance covered per minute (m·min^−1^), walking (0–7 km·h^−1^), jogging (7–14 km·h^−1^), running (14–21 km·h^−1^), sprinting (>21 km·h^−1^), high-intensity activity (percentage of total distance traveled up to 16 km·h^−1^), average-speed walking (km·h^−1^), total accelerations (m·s^−2^, n·min^−1^), low accelerations (1–2.5 m·s^−2^, n·min^−1^), medium accelerations (2.5–4 m·s^−2^, n/min), high accelerations (>4 m·s^−2^, n·min^−1^), total decelerations (m·s^−2^, n·min^−1^), low decelerations (−1–2.5 m·s^−2^, n/min), medium decelerations (−2.5–4 m·s^−2^, n·min^−1^), high accelerations (-(>4 m·s^−2^, n·min^−1^), metabolic power (W·kg^−1^ per minute), PlayerLoad·min^−1^ (a.u.), total impacts per minute, very low impacts (5–6 G per minute), low impacts (6–6.5 G per minute), medium impacts (6.5–7 G per minute), high impacts (7–8 G per minute), very high impacts (8–10 G per minute), severe impacts (>10 G per minute)
Duthie et al. (2018) [30]	N = 96, elite junior soccer players (U15, U16, U17); 2025/2016/2017 seasons, 61 games (441 individual match observations)	match	STATSport VIPER Units/Viper pods, 10 Hz (Belfast, UK)	speed (distance covered per unit of time (m·s^−1^), acceleration (m·s^−2^), metabolic power (W·kg^−1^)
Doncaster et al. (2019) [31]	N = 17, highly trained youth soccer players; 2-week period, 3 matches	match	Catapult (N/A) unit, 10 Hz (Melbourne, Australia)	total distance (m), meters per minute (m·min^−1^), relative high-speed running distance (m), relative high-speed efforts (n), relative very high-speed running distance (m), very high-speed efforts (n), relative sprint distance
Aquino et al. (2019) [55]	N = 51, youth soccer players (U11, U13, U15, U17, U20)	training + match	Sports^®^ QSTARZ, 5 Hz (Taipei, Taiwan)	total distance covered (m), high-intensity accelerations (>2 m·s^−2^, m), high-intensity decelerations (≤−2 m·s^−2^, m), high-intensity running (60.1 to 100% of maximum running speed)
Doncaster et al. (2020) [32]	N = 29, professional soccer outfield players (U23); full season, 17 competitive matches (130 separate physical match profiles)	match	STATSport VIPER Units/Viper pods, 10 Hz (Belfast, UK)	total distance (m), high-speed running (≥19.8 km·h^−1^, m), metabolic power (>25.5 W·Kg^−1^, below 19.8 km·h^−1^, m)
Marynowicz et al. (2020) [24]	N = 18, elite male youth soccer players; 18-week in-season; 804 training observations	training	PLAYERTEK, 10 Hz, integrated IMU 400 Hz (Dundalk, Ireland)	total duration (minutes), total running distance covered (m) (+/min), player load (a.u.), high-speed-running distance (>19.8 km·h^−1^, m) (+/min), impacts (n) (+/min), accelerations (>2 m·s^−2^, n) (+/min), decelerations (<−2 m·s^−2^, n) (+/min)
Wass et al. (2020) [33]	N = 25, academy soccer players (U18); 7 professional development league matches	match	STATSport VIPER Units/Viper pods, 18 Hz, integrated IMU 100 Hz (Belfast, UK)	meters per minute (m·min^−1^), high metabolic load distance per minute (accelerating over 2.5 m·s^−2^, sprinting over 5.5 m·s^−1^; m), high speed running per minute (>5 m·s^−1^; m), accelerations per minute (>3 m·s^−2^; n), decelerations per minute (>3 m·s^−2^; n)
Scantlebury et al. (2020) [34]	N = 8, adolescent male soccer players; 14-week	training	Catapult Optimeye S5, 10 Hz, integrated IMU 100 Hz (Melbourne, Australia)	total distance (m), lower-intensity running (m), higher-intensity running (m), PlayerLoad (a.u.), PlayerLoad_slow_ (a.u.)
Sánchez-Sánchez et al. (2021) [35]	N = 17, junior soccer players; 11-week	training + match	GPSports System SPI Pro, 15 Hz (Canberra, Australia)	total distance (m), high-intensity distance (>19.1 km·h^−1^, m), total sprint distance (>25.1 km·h^−1^, m, n), repeated sprint (>25.1 km·h^−1^, n), acceleration (>2.75 m·s^−2^, n)
Kovacs et al. (2021) [36]	N = 112, soccer players across 12 clubs (U17); 1-month tournament, 22 matches data	match	Catapult Optimeye S5, 10 Hz, integrated IMU 100 Hz (Melbourne, Australia)	total distance (m), low-speed running distance (<14.4 km·h^−1^, m), high-speed running (>14.4 km·h^−1^, m), very-high-speed running distance (>19.8 km·h^−1^, m), sprint distance (>25.2 km·h^−1^, m), PlayerLoad^TM^ (a.u.)
Parr et al. (2021) [37]	N = 37, elite male youth soccer players from one professional soccer academy (U14, U15, U16); full season, 30 matches (274 player files)	match	STATSport VIPER Units/Viper pods, 10 Hz, integrated IMU 100 Hz (Belfast, UK)	total distance, high-speed running distance (≥5.5 m·s^−1^, m), very-high-speed running distance (≥7.0 m·s^−1^, m), maximum speed (km·h^−1^), accelerations (>3.0 m·s^−2^, n)
Maughan et al. (2021) [38]	N = 22, professional youth soccer players; 46-week in-season with 6-week pre-season and two competitive seasons	training + match	Catapult Optimeye X4 GPS 10 Hz, integrated IMU 10–100 Hz (Melbourne, Australia)	total duration (minutes), total distance (m) (+/min), PlayerLoad^TM^ (a.u.) (+/min), low-intensity running (<14.4 km·h^−1^, m) (+/min), running (19.8–24.98 km·h^−1^, m), sprinting (>24.98 km·h^−1^, m) (+/min), accelerations (>2 m·s^−2^, count) (+/min), decelerations (<−2 m·s^−2^, count) (+/min)
Muñoz-Castellanos et al. (2022) [39]	N = 20, male youth soccer players selected from one Spanish professional academy from 3 age categories (U14, U16, U19); one season, 21 matches (60 player match observations)	match	Catapult (N/A) unit, 10 Hz, integrated IMU 100 Hz (Melbourne, Australia)	total distance covered (m) (+/min), distance covered while running at high speeds (18.0–20.9 km·h^−1^, m) (+/min), distance covered while running at very high speeds (21.0–23.9 km·h^−1^, m) (+/min), distance covered while sprinting (>24.0 km·h^−1^, m) (+/min), high accelerations (>2.5 m·s^−2^, n) (+/min), high decelerations (<2.5 m·s^−2^, n) (+/min), peak speed (km·h^−1^)
Teixeira et al. (2022) [8]	N = 60, elite male youth soccer players U15, U17, U19; 6 weeks; 324 observations (18 trainings)	training	STATSports Apex, 18 Hz, integrated IMU 100 Hz (Belfast, UK)	total duration (minutes), total distance covered (m), average speed (m·min^−1^) maximal running speed (m·s^−1^), relative high-speed running (19.8–25.1 km·h^−1^, m), high-metabolic-load distance (25.5 W·kg^−1^, m), sprint distance (m), sprint efforts (n), maximal running speed (m·min^−1^), dynamic stress load (a.u.), accelerations (>3 m·s^−2^, n), decelerations (≤3 m·s^−2^, n)
Maughan et al. (2022) [40]	N = 20, male professional youth soccer players; 47-week season (competition 1—autumn and 2—spring); 3207 individual recordings (695 matches, 2512 training sessions)	match	Catapult Optimeye X4, 10 Hz, integrated IMU 10 Hz to 100 Hz (Melbourne, Australia)	total distance (m), PlayerLoad (a.u.), low-intensity running (<14.4 km·h^−1^, m), high-speed running (19.8–24.98 km·h^−1^, m), sprinting distance (24.98 km·h^−1^, m), accelerations (>2 m·s^−2^, count), decelerations (>−2 m·s^−2^, count)
Kunzmann et al. (2022) [41]	N = 66, soccer player (U17, U19); spring national season; 12 highest-level match)	match	GPSport Team AMS, 15 Hz, integrated IMU 100 Hz (not published, but variables tend to say that) (Canberra, Australia)	total distance covered (m), relative total distance covered (m), distance/time (m·min^−1^), maximal running sprint (km·h^−1^), average speed (km·h^−1^), distance covered in different intensity speed zones: Z1 (0–0.7 km·h^−1^, m), Z2 (0.7–7.2 km·h^−1^, m), Z3 (7.2–14.4 km·h^−1^, m), Z4 (14.4–19.8 km·h^−1^, m), Z5 (19.8–25.2 km·h^−1^, m), Z6 (>25.2 km·h^−1^, m), high metabolic load distance, very high magnitude of acceleration Z3 (>3.6 m·s^−2^; n), high magnitude of acceleration Z2 (2.4–3.6 m·s^−2^; n), low magnitude of acceleration Z1 (<2.4 m·s^−2^; n), very high magnitude of deceleration Z3 (<−3.6 m·s^−2^; n), high magnitude of deceleration Z2 (−2.4–−3.6 m·s^−2^; n), low magnitude of deceleration Z1 (<−2.4 m·s^−2^; n)
Szigeti et al. (2022) [42]	N = 84, elite national soccer players (U-17); 64 matches	match	Catapult S5, S7 Vector, GPS 10 Hz, integrated IMU 100 Hz (Melbourne, Australia)	total duration (minutes), total distance (m), meterage per minute (m·min^−1^), total player load (a.u.), high-intensity running distance (>19.8 km·h^−1^, m), sprint distance (>25.2 km·h^−1^, m), maximum sprint speed distance (>30 km·h^−1^, m), explosive distance (>2 m·s^−2^, m), accelerations (>3 m·s^−2^, n), decelerations (<−3 m·s^−2^, n), total inertial movement analysis (a.u.)
de Dios-Álvarez et al. (2023) [25]	N = 21, professional academy soccer players	training + match	PLAYERTEK, 10 Hz, integrated IMU 400 Hz (Dundalk, Ireland)	total distance covered (m), running distance (14–21 km·h^−1^, m), high-speed distance (>21 km·h^−1^, m), sprint distance (>24 km·h^−1^, m), total accelerations and decelerations (n), low-/medium-/high-/very-high-intensity accelerations (1–2 m·s^−2^/2–3 m·s^−2^/3–4 m·s^−2^/>4 m·s^−2^, m), low-/medium-/high-/very-high- intensity decelerations (−1–−2 m·s^−2^/−2–−3 m·s^−2^/−3–−4 m·s^−2^/<−4 m·s^−2^, m), high metabolic power (>20 W·kg^−1^, m), player load (a.u.)
Douchet et al. (2023) [44]	N = 23, elite male academy soccer players (U17–U19); 20 competitive weeks; 738 individual training sessions and 123 individual matches	training + match	Fieldwizz, 10 Hz, integrated IMU 100 Hz (Lausanne, Switzerland)	total distance, distance at moderate speed (15–20 km·h^−1^, m), distance at high speed (20–25 km·h^−1^, m), sprint (>25 km·h^−1^, m), total number of accelerations (>3 m·s^−2^, n), decelerations (<−3 m·s^−2^, n)
Marynowicz et al. (2023) [46]	N = 18, youth soccer players; 18-week in-season; 804 training observations	training	PLAYERTEK, 10 Hz, integrated IMU 400 Hz (Dundalk, Ireland)	total distance (m) (+/min), player load (a.u.) (+/min), high-speed running distance (>19.8 km·h^−1^, m) (+/min), accelerations (>2 m·s^−2^, n) (+/min)
Teixeira et al. (2023) [47]	N = 60, sub elite youth male soccer academy players U15, U17, U19; 6-week; 324 observation (18 trainings)	training	STATSports Apex 18 Hz, integrated IMU 10–100 Hz (Belfast, UK)	total distance (m), average running velocity (m·min^−1^, m), maximal running speed (m·s^−1^), maximal speed per second (m·s^−1^), relative high-speed running distance (19.8–25.1 km·h^−1^, m), sprint (>25.1 km·h^−1^, n), average sprint distance (>25.1 km·h^−1^, m), high metabolic load distance (>25.5 W·kg^−1^, m), dynamic stress load (a.u.), accelerations (>3 m·s^−2^, n), decelerations (<3 m·s^−2^, n)
Castillo-Rodríguez et al. (2023) [57]	N = 15, soccer players (U19); 5 weeks; four training sessions and four official matches	training + match	GPSport SPI-PRO, 5 Hz (Canberra, Australia)	total distance (m), walking (0.1–6.9 km·h^−1^, m), low-intensity running (7.0–12.9 km·h^−1^, m), medium-intensity running (13.0–17.9 km·h^−1^, m), high-intensity running (18.0–20.9 km·h^−1^, m), sprinting (>21.0 km·h^−1^, m)
de Dios-Álvarez et al. (2024) [43]	N = 60, youth soccer players (U16, U19); two competition seasons, 978 observations from small-sided games during training, 62 observations from matches (10 matches)	training + match	PLAYERTEK, 10 Hz, integrated IMU 400 Hz (Dundalk, Ireland)	total distance covered (m), total distance covered relative (m), high-speed distance (18–21 km·h^−1^, m), very-high-speed distance (21–25 km·h^−1^, m), sprint distance (>25 km·h^−1^, m), relative sprint distance (m), accelerations (>3 m·s^−2^, n) (+/min), decelerations (<−3 m·s^−2^, n) (+/min), high metabolic power (>20 W·kg^−1^, n), player load (a.u.)
Kanaras et al. (2024) [54]	N = 20, elite young male soccer players (U17); 18 microcycles	training + match	STATSports Apex 18 Hz, integrated IMU 10–100 Hz (Belfast, UK)	total distance (m), high-speed running distance (19.8–25.2 km·h^−1^, m), zone 6 distance covered (>25.2 km·h^−1^, m), accelerations (>2 m·s^−2^, n), decelerations (≥−2 m·s^−2^, n)
Teixeira et al. (2024) [45]	N = 60, male youth football academy players (U15, U17, U19); 6-week period	training	STATSports Apex 18 Hz, integrated IMU 10–100 Hz (Belfast, UK)	number of accelerations (>3 m·s^−2^, n), number of decelerations (<3 m·s^−2^, n), dynamic stress load
Havanecz et al. (2025) [26]	N = 50, male elite youth academy soccer players U15/U17/U19; 11-week in-season; 1386 observations (145 trainings, 33 matches)	training + match	Catapult Vector S7, GPS 10 Hz, integrated IMU 100 Hz (Melbourne, Australia)	total duration (minutes), total distance covered (m) (+/min), medium-speed running (14.4–19.7 km·h^−1^, m) (+/min), high-speed running (19.8–25.1 km·h^−1^, m) (+/min), sprint distance (>25.2 km·h^−1^, m) (+/min), accelerations (>1.5 m·s^−2^, m) (+/min), decelerations (<−1.5 m·s^−2^, m) (+/min) inertial movement analysis (a.u.) (+/min), PlayerLoad^TM^ (a.u.) (+/min)
Havanecz et al. (2025) [27]	N = 19, male elite youth academy soccer players U16; 11-week in-season; 465 observations (50 trainings, 11 matches)			
Pérez-Contreras et al. (2025) [48]	N = 42, elite young football players (U15, U17, U20); one tournament 9 match data (144 observations)	match	Catapult Vector X7, 10 Hz, integrated IMU 100 Hz (Melbourne, Australia)	total distance covered (m), player load (a.u.), D7 (0–7 km·h^−1^, m), D13 (7–13 km·h^−1^, m), D19 (13–19 km·h^−1^, m), D23 (19–23 km·h^−1^, m), high-speed runs D23 (>23 km·h^−1^, n)
Tan et al. (2025) [50]	N = 46, male academy soccer players (U17); 2 weeks	training + match	Polar Team Pro, 10 Hz (Kempele, Finland)	total distance covered per minute (m·min^−1^), average speed (km·h^−1^), distance per minute covered in zone 4 (15.0–18.99 km·h^−1^, m), distance per minute covered in zone 5 (>19.0 km·h^−1^, m)
Fernández-Jávega et al. (2025) [49]	N = 53, young male soccer players (U14-U16); 8-week	training	Oliver IMU^®^, 10 Hz (Barcelona, Spain)	total distance (m), maximal velocity (km·h^−1^), total distance, walking (<6 km·h^−1^, m), low-intensity running (6–12 km·h^−1^, m), medium-intensity running (12–18 km·h^−1^, m), high-intensity running (>18 km·h^−1^, m), medium-intensity accelerations (2–3 m·s^−2^, m), high-intensity accelerations (>3 m·s^−2^, m), medium-intensity decelerations (<−2 to −3 m·s^−2^, m), high-intensity accelerations (<−3 m·s^−2^, m)

Abbreviations: m: meter; n: number (count); a.u.: arbitrary unit; min: minutes; MAS: maximal aerobic speed; ASR: anaerobic speed reserve; GPS: global positioning system; IMU: inertial measurement unit. Player load (PL) represents a manufacturer-specific arbitrary unit derived from triaxial accelerometer data.

**Table 2:** Summary of reported speed and acceleration threshold ranges across included studies.

**Variable**	**Reported Ranges**	**Example Thresholds Reported**
High-speed running	>14.4 to >21.0 km·h^−1^	>14.4; >18.0; 18.0–20.9; >19.1; 19.8–25.1; 19.8–25.2; 20–25; >21.0 km·h^−1^
Sprint distance	>21.0 to >25.2 km·h^−1^	>21.0; >24.0; >24.98; >25.0; >25.1; >25.2 km·h^−1^
Accelerations	>1.5 to >4.0 m·s^−2^	>1.5; >2.0; >2.5; >2.75; >3.0; >4.0 m·s^−2^
Decelerations	<−1.5 to <−4.0 m·s^−2^	<−1.5; <−2.0; <−2.5; <−3.0; <−4.0 m·s^−2^

## Data Availability

Not applicable.

## Abbreviations

The following abbreviations are used in this manuscript:

ACC	Acceleration
ASR	Anaerobic speed reserve
AU	Arbitrary unit
CV	Coefficient of variation
DEC	Deceleration
ETL	External training load
GNSS	Global navigation satellite system
GPS	Global positioning system
HDOP	Horizontal dilution of precision
HSR	High-speed running distance
IMU	Inertial measurement unit
ITL	Internal training load
MAS	Maximal aerobic speed
MSS	Maximal sprint speed
PHV	Peak height velocity
PL	Player load
SD	Sprint distance
TD	Total distance
TL	Training load
